# Increased epithelial thickness and reduced HIV receptor expression in the ectocervical mucosa is associated with relative HIV resistance

**DOI:** 10.1186/1742-4690-9-S2-P190

**Published:** 2012-09-13

**Authors:** K Broliden, J Kimani, B Ball, J Cheruiyot, N Mugo, W Jaoko, F Plummer, R Kaul, T Hirbod

**Affiliations:** 1 Karolinska Institutet, Stockholm, Sweden

## Background

The female genital tract is an important site of HIV acquisition, but the epithelial and submucosal tissue factors associated with HIV susceptibility have not been defined.

## Methods

Ectocervical biopsies were obtained from HIV-exposed seronegative (HESN) women (n=20) and HIV-seronegative lower risk controls (n=20). Epithelial thickness and tissue distribution of immunological markers were assessed in situ by immunohistochemistry and measurement of mRNA expression was performed by quantitative PCR.

## Results

The thickness of the ectocervical epithelium was significantly higher in HESN vs. lower risk subjects. CD4 and DC-SIGN mRNA expression was significantly lower in HESN than lower risk women, and in situ immunohistochemical analysis confirmed the reduced CD4 expression in HESN participants. In addition, immunohistochemistry demonstrated lower CCR5 and higher Langerin expression in the HESN subjects.

## Conclusion

A thicker epithelial barrier and altered expression of HIV binding receptors in the ectocervix of HESN women may contribute to protection against HIV transmission.

